# DAILY NEGATIVE AND POSITIVE EVENTS AND DAILY WELL-BEING: DYADIC LINKS IN COUPLES LIVING WITH EARLY-STAGE DEMENTIA

**DOI:** 10.1093/geroni/igad104.1764

**Published:** 2023-12-21

**Authors:** Courtney Polenick, Diarratou Kaba, Nikita Daniel, Olivia Lambert, Shreya Salwi, Juhi Valera, Kira Birditt

**Affiliations:** University of Michigan, Ann Arbor, Michigan, United States; University of Michigan, Ann Arbor, Michigan, United States; University of Michigan, Ann Arbor, Michigan, United States; University of Michigan, Ann Arbor, Michigan, United States; University of Michigan, Ann Arbor, Michigan, United States; University of Michigan, Ann Arbor, Michigan, United States; Institute for Social Research, University of Michigan, Ann Arbor, Michigan, United States

## Abstract

Spouses have powerful influences on well-being in middle and later life, but we know little about how daily events impact well-being in older couples that include persons living with dementia (PLWD). We examined associations between daily negative and positive events and daily well-being (negative mood and positive mood) in 36 heterosexual married couples living with early-stage dementia. Participants included PLWD (M = 71.47 years, SD = 6.90, range = 53-82 years) and their spouses (M = 69.69 years, SD = 7.26, range = 59-87 years) who both completed brief morning and evening phone interviews over 7 consecutive days. Longitudinal actor-partner interdependence models controlled for own and partner reports of daily well-being in the previous day, whether each day was a weekend day, and the gender of PLWD. We estimated separate models for negative mood and positive mood. Both PLWD and their spouses reported higher negative mood when they reported more negative events in the same day and when their partner reported more negative events in the previous day. Over and above these associations, both partners in the couple reported lower negative mood on days when spouses of PLWD reported more positive events. Spouses of PLWD reported higher positive mood on days when they reported more positive events, but lower positive mood on days when PLWD reported more positive events. These findings highlight the importance of understanding dyadic influences on daily well-being among PLWD and their spouses.

